# Diversity of Free-Living Amoebae in New Zealand Groundwater and Their Ability to Feed on *Legionella pneumophila*

**DOI:** 10.3390/pathogens13080665

**Published:** 2024-08-07

**Authors:** Sujani Ariyadasa, Sophie van Hamelsveld, William Taylor, Susan Lin, Panan Sitthirit, Liping Pang, Craig Billington, Louise Weaver

**Affiliations:** Institute of Environmental Science and Research, 27 Creyke Road, Ilam, Christchurch 8041, New Zealandliping.pang@esr.cri.nz (L.P.);

**Keywords:** free-living amoebae, groundwater, *Legionella pneumophila*, 18S amplicon sequencing, metagenomics, co-culture, amoebae-mediated pathogen dispersal

## Abstract

Free-living amoebae (FLA) are common in both natural and engineered freshwater ecosystems. They play important roles in biofilm control and contaminant removal through the predation of bacteria and other taxa. Bacterial predation by FLA is also thought to contribute to pathogen dispersal and infectious disease transmission in freshwater environments via the egestion of viable bacteria. Despite their importance in shaping freshwater microbial communities, the diversity and function of FLA in many freshwater ecosystems are poorly understood. In this study, we isolated and characterized FLA from two groundwater sites in Canterbury, New Zealand using microbiological, microscopic, and molecular techniques. Different methods for groundwater FLA isolation and enrichment were trialed and optimized. The ability of these isolated FLA to predate on human pathogen *Legionella pneumophila* was assessed. FLA were identified by 18S metagenomic amplicon sequencing. Our study showed that *Acanthamoeba* spp. (including *A. polyphaga*) and *Vermamoeba veriformis* were the main FLA species present in both groundwater sites examined. While most of the isolated FLA co-existed with *L. pneumophila*, the FLA populations in the *L. pneumophila* co-culture experiments predominantly consisted of *A. polyphaga*, *Acanthamoeba* spp., *Naegleria* spp., *V. vermiformis*, *Paravahlkampfia* spp., and *Echinamoeba* spp. These observations suggest that FLA may have the potential to act as reservoirs for *L. pneumophila* in Canterbury, New Zealand groundwater systems and could be introduced into the local drinking water infrastructure, where they may promote the survival, multiplication, and dissemination of *Legionella*. This research addresses an important gap in our understanding of FLA-mediated pathogen dispersal in freshwater ecosystems.

## 1. Introduction

Free-living amoebae (FLA) are a diverse group of protozoa universally found in natural freshwater and terrestrial ecosystems [1]. They can also proliferate in engineered water systems (EWS); previous work has described FLA in chlorinated tap water [2], drinking water storage tanks [3], cooling towers [4], recreational water [5], and hospital hot water systems [6]. There is also evidence indicating the presence of FLA in groundwater [5,7]. Under favorable environmental conditions, FLA exist in their metabolically active trophozoite state, feeding on bacteria, fungi, and algae via phagocytosis [8]. As dominant environmental predators, FLA play important roles in regulating microbial communities and the dispersal of pathogenic bacteria in freshwater ecosystems [9]. Studies have shown that the selective predation pressure of FLA may also favor the emergence of resistance mechanisms among bacteria [10]. In EWS, encysted forms of FLA are highly resistant to disinfection treatment, thereby protecting phagocytosed bacteria from environmental stressors and facilitating their re-emergence when conditions are favorable [8]. Despite their importance in microbial ecology and environmental health, the diversity and function of FLA in many freshwater ecosystems are poorly understood, particularly in New Zealand groundwater. Understanding FLA diversity in groundwater is important for safeguarding public and environmental health as it is a primary source of the drinking water in many parts of New Zealand and the world [11,12].

It is well-established that bacteria may resist predation by FLA, becoming engulfed by feeding amoebae but not undergoing lysis. Many important human pathogens, including *Legionella*, *Pseudomonas*, *Mycobacterium*, and *Burkholderia*, have been identified to be amoeba-resistant [8]. Once internalized, the amoeba-resistant bacteria may remain viable for much longer than in their planktonic state [9] as they can exploit the encystation process of FLA to protect themselves from unfavorable environmental conditions, resuming replication when the FLA resume normal metabolic activity. It is speculated that FLA predation may contribute to the development of bacterial resistance to human macrophages due to the similarities in the predation processes between FLA and macrophages [13]. There is also evidence that FLA may act as a “training ground” for both established and emerging amoeba-resistant microbes, priming them for more robust human infection [14]. For example, *L. pneumophila* replication within FLA is known to enhance its virulence (infection and replication within human monocytes) [15] and resistance to chlorine, temperature, salt, and acidity [16]. Similarly, disinfectant-treated *L. pneumophila* in viable but non-culturable states has been revived to resume an infective state via co-culturing with FLA [17]. It is suggested that the increase in *L. pneumophila* virulence following intracellular replication could compensate for their low infectious doses in EWS sources typically linked with legionellosis outbreaks [18]. At least 20 species of FLA, including *Acanthamoeba* spp., *Hartmannella* spp., and *Naegleria* spp., have been characterized as environmental hosts for *L. pneumophila* [19]. As such, *L. pneumophila* levels in EWS are linked to FLA species and their abundance in these water systems [20].

In this preliminary study, we isolated and characterized FLA from two groundwater sites in Canterbury, New Zealand. The isolation of FLA from environmental samples can be difficult. Rich media are prone to bacterial and fungal contamination, while amoebic FLA are themselves predated upon by other protozoa that may be co-isolated from environmental samples. To address these challenges, we trialed and optimized different methods for groundwater FLA enrichment and the removal of non-target protozoa using lawns of *E. coli* as the principal food source. The ability of isolated FLA to predate on the human pathogen *L. pneumophila* was assessed using co-culture as amoeba internalization is a critical step in *L. pneumophila* intracellular replication, dispersal, and persistence in natural freshwater environments and EWS. Subsequently, metagenomic amplicon sequencing of the 18S rRNA gene was performed on DNA extracted from populations of FLA that were enriched via co-culturing with predation on *E. coli* and/or *L. pneumophila*.

## 2. Materials and Methods

### 2.1. Media and Chemicals

*E. coli* was cultured in Luria–Bertani (LB) broth and agar (Invitrogen, Waltham, MA, USA). *L. pneumoniae* was cultured on buffered charcoal yeast extract (BCYE) agar (Oxoid, Thermo Fisher Scientific, North Shore City, New Zealand) and in buffered yeast extract (BYE) broth (Oxoid, Thermo Fisher Scientific, North Shore City, New Zealand). Non-nutritive agar (NNA) composed of 15 g/L bacteriological agar, 4.6 mM Na_2_HPO_4_, and 2.9 mM KH_2_PO_4_ was used for isolating and culturing amoebae from groundwater samples. Page’s amoebic saline (PAS) solution composed of 120 mg/L NaCl, 4 mg/L MgSO_4_.7H_2_O, 4 mg/L CaCl_2_ · 2H_2_O, 142 mg/L Na_2_HPO_4_, and 136 mg/L KH_2_PO_4_ was used to apply bacteria for feeding amoebae and for making bacterial lawns [21]. Phosphate buffered saline (PBS; Oxoid) was used for washing bacterial cultures.

### 2.2. Bacterial Strains

*E. coli* strain ATCC25922 was used for isolation and feeding of amoebae from groundwater samples. *L. pneumophila* strain Philadelphia 1 (ATCC33152) was used for amoebae migration fronts and feeding trials.

### 2.3. Groundwater Sampling Locations

Groundwater was collected from two wells in Canterbury, New Zealand in March 2023. Each site was only sampled once as a part of an exploratory study conducted to understand the FLA diversity in the groundwater of Canterbury, New Zealand. The first sampling location was at a rural study site in North Canterbury, New Zealand (site A). Sampling site A is surrounded by mixed land use of a predominately agricultural nature but with nearby urban and industrial activity. The average temperature, dissolved oxygen, conductivity, and pH of the groundwater collected from sampling site A were 15.5 (±0.2) °C, 1.1 (±0.5) mg/L, 197.0 (±4.5) µS/cm, and 6.08 (±0.1), respectively. The second site was a drinking water well that services a large university (site B). The surrounding land use is predominately urban.

### 2.4. Groundwater Sampling Procedure

Eight 2.5 L groundwater samples were collected from each of the two sites at a depth of 4.5 m. The temperature and pH of samples were recorded at the time of sampling. Both wells were purged for approximately five minutes prior to sample collection. Samples were transported on ice and processed within four hours of collection.

### 2.5. Isolation of Free-Living Protozoa from Groundwater

Protozoa were isolated from groundwater samples by membrane filtration and grown on *E. coli* lawns [3]. Lawns were prepared as follows: 50 mL cultures of *E. coli* were grown for 16–18 h in LB broth at 37 °C with aeration to achieve a concentration of approximately 1 × 10^9^ CFU mL^−1^. Broth cultures were washed twice in an equal volume of PAS at 4 °C before being resuspended in 10 mL PAS and kept at 4 °C. Further, 200 µL aliquots of *E. coli* suspended in PAS were aseptically spread onto NNA plates (*E. coli*–NNA) to achieve a concentration of approximately 2 × 10^8^ CFU mL^−1^ per plate before drying under sterile conditions. For isolation from groundwater samples, 1 L aliquots of water samples were passed through nitrocellulose filters (Whatman, Cytiva, Marlborough, MA, USA) under vacuum. This resulted in 20 samples per sampling location. Four filter pore sizes were used: 0.1, 0.22, 0.45, and 1 µm. Each water aliquot was passed through only one filter size. We laid one filter per plate onto the prepared *E. coli* lawns. Using a Pasteur pipette, we added sterile PAS to the filter, dropwise, until the plate surface was saturated [22]. Plates were placed in sealed containers, incubated at 20 °C to promote FLA activity, and observed every 2–3 days under an inverted microscope (Nikon Ts2, Coherant Scientific, Thebarton, Australia) to observe the FLA “migration fronts” or clearance lines spreading outwards from the filter paper [23]. Plates containing visible “migration fronts” were frequently “fed” by dropwise application of 100 µL freshly prepared PAS–*E. coli* suspension.

### 2.6. Isolation of Amoebae from Groundwater Fauna

Different methods were trialed for isolation of FLA trophozoites from other protozoa fauna and their propagation. Isolation methods trialed were (1) agar block transfer (“wet” and/or “dry”), (2) serial dilution, (3) direct trophozoite harvesting, and (4) transfer of encysted trophozoites. In the agar block transfer method, a block of agar surrounding the trophozoite was cut using a sterile blade under the microscope and placed on a fresh *E. coli* lawn on NNA [24]. The plates were either periodically saturated with PAS (wet transfer) or maintained without liquid (dry transfer). In serial dilution method, trophozoites were scraped from the *E. coli* lawns and serially diluted in PAS in 12-well plates up to a maximum dilution factor of 10. Direct trophozoite harvesting involved aspirating single trophozoites with a small volume of PAS from the isolation plate and transferring onto fresh *E. coli* lawns while observing under the microscope. In encysted trophozoite transfer, active trophozoites isolated using methods 1, 2, or 3 were maintained at 20 °C without feeding for 14–21 days to promote encystation. Trophozoite encystation was confirmed by microscopy; double-walled cysts were picked using sterile tweezers and placed on fresh *E. coli* lawns to promote further propagation.

### 2.7. Feeding Experiments

Feeding experiments were conducted using *L. pneumophila* as a model for amoeba-resistant opportunistic waterborne bacteria. Prior to feeding experiments, *L. pneumophila* was grown to saturation in buffered charcoal yeast broth at 37 °C 121 rpm, stationary phase cells harvested, cleaned, resuspended in PAS, and spread on NNA plates to establish lawns for feeding experiments. Groundwater FLA propagated on *E. coli* lawns were introduced onto the pathogenic bacterial lawns by agar block transfer method. Plates were observed under the microscope every 2–3 days for trophozoite feeding behavior. In both *E. coli* and *L. pneumophila* experiments, plates containing actively feeding FLA were incubated until the trophozoites formed monolayers on bacterial lawns. Monolayers were passaged three times on fresh *E. coli* or *L. pneumophila* bacterial lawns before final harvesting. Each monolayer was harvested using a sterile inoculation loop and resuspended in PAS. Monolayers from triplicate plates were combined to obtain a FLP pellet sufficient for DNA extraction.

### 2.8. DNA Extraction and PCR

DNA from presumptive amoeba cultures was extracted using the Qiagen Blood and Tissue Kit (Qiagen, Hilden, Germany) according to the manufacturer’s instructions. DNA extracts were quantified by fluorometry (Qubit, Thermo Fisher Scientific, North Shore City, New Zealand) and checked for purity by spectrophotometry (Nanodrop, Thermo Fisher Scientific, North Shore City, New Zealand). DNA extracts were amplified using forward and reverse primers 5′CAGCAGCCGCGGTAATTCC3′ and 5′CCCGTGTTGAGTCAAATTAAGC3′, which targets a 650 bp segment of the V4–V5 region of eukaryotic 18S rRNA gene [25]. Thermal cycling conditions used for the amplification of 18S gene of amoebae are outlined in Table S1. PCR products were visualized using MultiNA to rule out bacterial genomic DNA amplification and quantified using spectrophotometry.

### 2.9. DNA Sequencing and Bioinformatic Analyses

PCR products were prepared for sequencing (cleaned, end-repaired, ligated with barcodes and adapters, and loaded onto a flow cell) from 16 samples using the Native Barcoding Kit 24 V14 (SQK-NBD114.24) protocol according to the manufacturer’s instructions. The sequencing library was loaded onto a R10.4.1 flowcell and sequenced on a GridION (Oxford Nanopore Technologies, Oxford Science Park, Oxford, UK) for 24 h. Raw reads were basecalled and had adapters removed using guppy (v6.0.1) and a super-accurate basecalling model (dna_r10.4.1_e8.2_400bps_sup-v4.1.0), and quality control was performed using pycoQC [26] (v2.5.0.3). Reads with a mean quality score > 12 had adapter sequences removed using cutadapt (v3.4) [27]. Reads shorter than 500 bp and longer than 800 bp were removed and cropped, respectively, using trimmomatic (v0.36) [28]. The remaining reads were taxonomically identified using emu (v3.4.5) and the PR^2^ database v5.0.0 [29,30].

## 3. Results and Discussion

**Figure 1:**
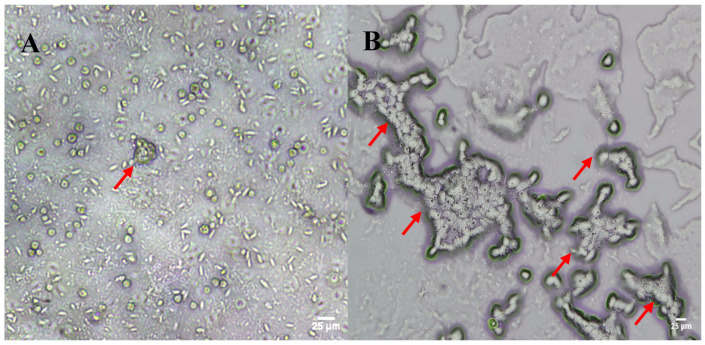
Inverted light microscopy images of FLA isolated from wet agar block transfer ((**A**), 40× magnification) and newly developed starvation isolation method ((**B**), 20× magnification). Red arrows point to FLA. Incorporating starvation to agar block transfer method reduced ciliate carryover and promoted FLA propagation.

Migration fronts were observed on all the *E. coli*–NNA plates containing filter papers in the first feeding trial, suggesting the presence of FLA in the groundwater samples collected from both sites. While this method promoted the emergence and proliferation of FLA within 3–5 days of culture establishment, it also facilitated the growth of large numbers of ciliates and some fungi (Figure 1A). FLA trophozoites grew slowly compared to the ciliates and fungi in the culture and appeared to be outcompeted for nutrition by the faster-growing microflora, resulting in a gradual decrease in their numbers. Ciliate overgrowth also interfered with our efforts to establish FLA subcultures through both the agar block and direct trophozoite transfer methods.

To promote FLA proliferation and reduce ciliate contamination in the FLA subcultures, we used a starvation step following the initial agar block, or direct trophozoite, transfer where the *E. coli*–NNA plates with presumptive positive FLA were left without supplementing PAS over two to three weeks to induce encystation. It is known that the majority of FLA species typically respond to nutrient exhaustion through encystation [31], whereas a lack of nutrition results in population decline and even total mortality in most species of ciliates [32]. While some ciliates form globular, ellipsoidal, or flask-like cysts, they can be easily morphologically distinguished from spherical or oval-shaped double-walled FLA cysts that are ~8–20 µm in diameter [33,34]. Starvation resulted in a reduction in ciliate numbers from 389 ± 59 ciliates per field before starvation to 109 ± 25 ciliates per field following starvation. From these plates, agar blocks surrounding cysts of presumptive positive FLA (~8–20 µm in diameter) were transferred onto fresh NNA plates containing *E. coli* and live *L. pneumophila* lawns. The monolayers of presumptive FLA obtained via the combined agar block transfer and starvation method are shown in Figure 1B. Preliminary microscopic observations confirmed that these monolayers consisted of a few FLA species. However, the FLA observed in these monolayers were not morphologically differentiated nor established as monoxenic cultures as our aim was determining the total diversity of the FLA in the groundwater samples collected.

**Figure 2:**
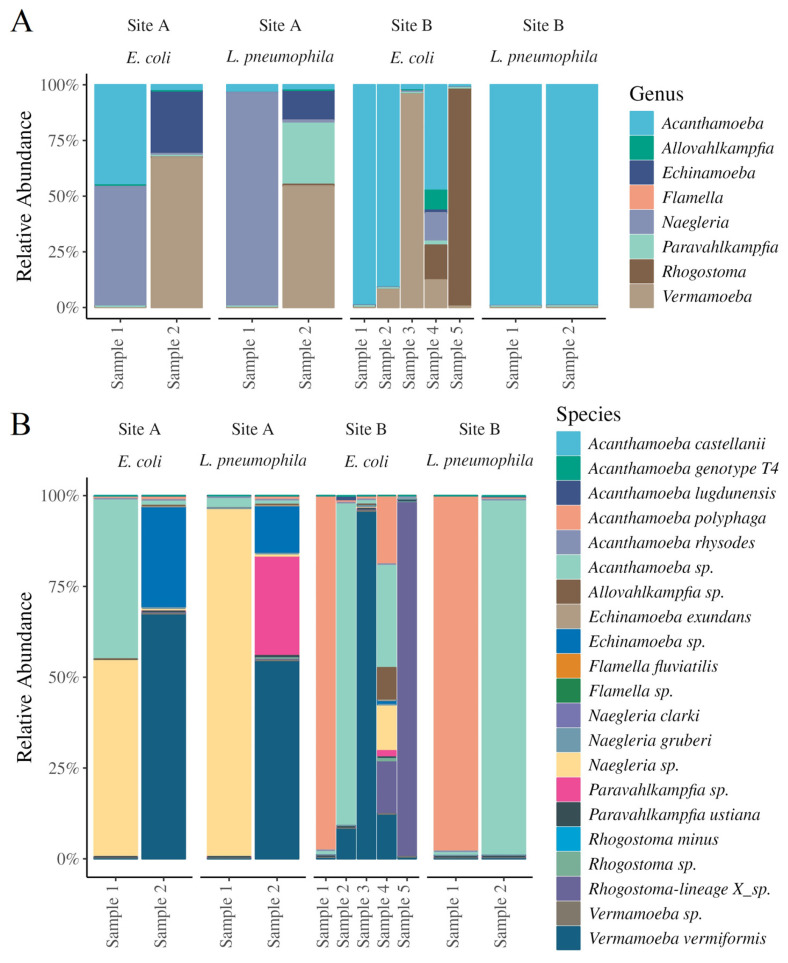
Relative abundances of FLA isolated from groundwater sites (site A, left; site B, right) in *E. coli* and *L. pneumophila* feeding experiments at genus level (**A**) and species level (**B**). Two samples were analyzed, except in the case of the ‘Site B, *E coli*’ samples, where five were used for the analysis.

The FLA genera and species, and their relative abundances, were identified via co-culture enrichment, PCR amplification of 18S rRNA, long-read amplicon sequencing, and taxonomic assignment using reference databases (Figure 2A,B). There were eight FLA genera identified with public health and ecological significance in the groundwater collected from the two sites: *Acanthamoeba*, *Allovahlkampfia*, *Echinamoeba*, *Flamella*, *Naegleria*, *Paravahlkamfia*, *Rhogostoma*, and *Vermamoeba* (Figure 2A). The FLA isolates identified as *Acanthamoeba* in the groundwater sites comprised *A. castellanii*, *A. genotype* T4, *A. lugdunensis*, *A. polyphaga*, *A. rhysodes*, and other *Acanthamoeba* species (Figure 2B). *Acanthamoeba* were isolated from both groundwater sampling sites and predated upon both *L. pneumophila* and *E. coli.* For example, in *E. coli* feeding experiments, *Acanthamoeba* spp. accounted for 44%, 89%, and 29% of the FLA relative abundances in composite sample 1 from site A and composite samples 3 and 4 from site B, respectively (Figure 2B). Similarly, *A. polyphaga* constituted 97% and 19% of the relative abundances in composite samples 1 and 4 of site B. *Acanthamoeba* were also predominant in the *L. pneumophila* feeding experiments conducted using FLA isolated from site B (Figure 2B). The high relative abundance of *Acanthamoeba* in the two groundwater sites is consistent with their frequent occurrence and wide dispersal in water and soil environments [35]. In addition, numerous previous investigations have reported the presence of *Acanthamoeba* in aquifer environments [36,37], groundwater [38,39], drinking water distribution systems [3,40,41], and recreational water [42].

*Vermamoeba vermiformis* (formerly *Hartmannella vermiformis*), a relatively understudied thermotolerant environmental FLA species commonly associated with hot water systems [43], was another key FLA species isolated from both groundwater sites. In *E. coli* feeding experiments, the relative abundance of *V. vermiformis* was 70% and 96% in groundwater sites A and B, composite samples 1 and 2, respectively. It was also detected in lower abundances (~10%) in composite samples 3 and 4 from site B. In *L. pneumophila* feeding experiments, only site A composite sample 2 indicated a high *V. vermiformis* abundance (Figure 2B). Similar to *Acanthamoeba*, *Vermamoeba* species are common inhabitants of natural freshwater environments. They also occur in surface water at concentrations ranging from 5 to 75 copies/L [44]. In addition to natural aquatic ecosystems, *V. vermiformis* has frequently been isolated from hot and cold water systems in hospital settings [6,45], drinking water sources [39], swimming pools and recreational water [42], rainwater storage tanks [46], and chlorinated drinking water storage tanks [3]. Due to their thermotolerant nature, *V. vermiformis* are known to be a predominant member of FLA populations in hot water systems and have even been exclusively recovered from drinking water systems with a higher-than-average water temperature [47].

Another thermotolerant environmental FLA [47], *Naegleria*, constituted 54% of the FLA relative abundance in composite sample 1 from *E. coli* feeding experiments conducted using groundwater from sampling site A. In *L. pneumophila* feeding experiments, the FLA community of composite sample 1 from the same site almost entirely consisted of *Naegleria* spp. (relative abundance 96%) (Figure 2B). They were also detected in composite sample 4 from site B. While some members of this genus (*N. fowleri*) are known to cause fatal central nervous system infections in humans [48], the species *N. clarki* and *N. gruberi* isolated in our study are not known to be implicated in human disease and have been isolated from surface water in previous investigations [49,50].

The other dominant taxa of FLA identified included *Echinamoeba* spp., a thermophilic FLA previously isolated from geothermal springs in Aotearoa, New Zealand [51], and *Paravahlkampfia* spp. Both were detected in composite sample 2 from site A with average relative abundances of 28% and 0.1% in the *E. coli* feeding experiments, and 13% and 27% in the *L. pneumophila* feeding experiments (Figure 2). An increase in *Paravahlkampfia* in the presence of *L. pneumophila* suggests that the FLA are able to successfully co-exist in the presence of these bacteria [52].

We also detected *Rhogostoma* spp. (Figure 2), a thecate amoeba that frequently inhabits wastewater treatment plants [53]. While these amoebae are known symbiotic hosts of *L. pneumophila* and other potential waterborne pathogens in wastewater environments [53], the present study did not evaluate the ability of *Rhogostoma* spp. to graze on *L. pneumophila*.

Our data show that most of the FLA species isolated from groundwater samples, namely *Acathamoeba* spp., *A. polyphaga*, *Naegleria* spp., *V. vermiformis*, *Paravahlkamfia*, and *Echinamoeba*, successfully coexisted with *L. pneumophila* serogroup 1, strain Philadelphia 1, a major causative agent of legionellosis in humans [54]. Previous studies report that FLA species, particularly *Acanthamoeba*, *Naegleria*, and *V. vermiformis*, can function as environmental reservoirs that enable *L. pneumophila* proliferation and transmission in freshwater ecosystems [55]. While the fate of *L. pneumophila* when internalized by these FLA was not investigated in the present study, the literature suggests that the FLA–*L. pneumophila* interactions are not always restricted to antagonistic outcomes that result in host lysis following *L. pneumophila* replication. Some FLA hosts may not facilitate the intracellular replication of internalized *L. pneumophila* (serogroup 1, strain Philadelphia) [56]. However, these non-replication permissive isolates still enable the persistence of intracellular *L. pneumophila.* In addition, some FLA hosts release vacuoles of intracellularly replicated *L. pneumophila* [57]. Despite some differences in host amoeba–bacteria dynamics, FLA grazing seems to promote *Legionella* persistence in natural and engineered freshwater ecosystems. The evidence suggests that FLA concentration and composition may influence the *Legionellae* concentration in drinking water environments [56]. These findings confirm that the FLA species isolated from Canterbury groundwater sites could potentially be reservoirs for *L. pneumophila*. As groundwater is the main source of drinking water in Canterbury, these FLA could be introduced into the local EWS, where they may promote *Legionella* persistence and dissemination, resulting in an increased risk of Legionnaires’ disease.

In addition to promoting persistence and dissemination of *L. pneumophila* and other amoeba-resistant microbes in water environments [8], the FLA themselves pose a public health risk as some species are known human pathogens [58]. While the cases are rare, the majority of reported FLA-related infections have been linked with recreational water exposure [59]. A linear increase in the protozoa grazing rates has been observed with rising water temperatures, up to 31 °C in previous research, particularly over the summer months [60]. There is also evidence that the rising water temperature may result in an expansion in the geographical range of FLA [61]. With changing climate conditions favoring the persistence and dispersal of FLA in surface water, FLA-associated infections are likely to become more prevalent in the future with the progression of climate change [62]. Consequently, FLA grazing is likely to have more of an impact on the pathogen ecology, both in environmental dissemination of amoeba-resistant bacteria and microbe removal through predation.

## 4. Conclusions

The two Canterbury groundwater sampling sites included in this investigation harbored several FLA species of importance to public and environmental health, including three thermophilic species. Most of the FLA isolates demonstrated an ability to successfully predate on *L. pneumophila* in co-culture, suggesting their potential to function as *L. pneumophila* reservoirs in water environments. By generating fundamental knowledge on FLA diversity in Canterbury groundwater, our study has contributed to the increasing knowledge of the FLA diversity in groundwater and in Aotearoa, New Zealand’s aquatic environments and provided novel methods for FLA isolation and characterization. These findings provide new insights into understanding FLA diversity in source waters and their role in the transmission of climate-sensitive waterborne infections caused by opportunistic pathogens that occur naturally in freshwater ecosystems. Future work focused on understanding the role of these environmental FLA in the uptake, proliferation, and release of *L. pneumophila* in laboratory co-cultures and biofilms will enable deciphering the mechanisms of the bacteria’s propagation and dispersal in groundwater and EWS environments. Additionally, time series sampling covering a wider range of groundwater sites would provide comprehensive insights into any seasonal variations in the FLA diversity in these ecosystems.

## Data Availability

All sequencing reads are available from the Sequence Read Archive (SRA) under BioProject PRJNA1134268. Requests to access other data should be directed to the corresponding author.

## Supplementary Materials

The following supporting information can be downloaded at https://www.mdpi.com/article/10.3390/pathogens13080665/s1, Table S1: Thermocycler conditions used for amplification of free-living protozoa DNA.
