# Supplementary material for: Diversity of Free-Living Amoebae in New Zealand Groundwater and Their Ability to Feed on Legionella pneumophila
Source: Pathogens. 2024 Aug 7;13(8):665. doi: 10.3390/pathogens13080665 (PMC11357516; doi:10.3390/pathogens13080665)
Supplement: Supplementary file 1 [file pathogens-13-00665-s001.zip › pathogens-3102190-supplementary.pdf]

Supplementary Materials

Table S1. Thermocycler conditions used for amplification of free-living protozoa DNA.

**Cycling Step**	**Time and Temperature**	**Cycles**
Initial denaturation	95 °C for 5 min	1
Denaturation	95 °C for 45 sec	
Annealing	60 °C for 45 sec	35
Extension	72 °C for 1 min	
Final extension	72 °C for 10 min	1
Cooling	4 °C	
